# Long-term air pollution exposure is associated with higher incidence of ST-elevation myocardial infarction and in-hospital cardiogenic shock

**DOI:** 10.1038/s41598-024-55682-6

**Published:** 2024-02-29

**Authors:** Jinah Cha, Se Yeon Choi, Seung-Woon Rha, Byoung Geol Choi, Jae Kyeong Byun, Sujin Hyun, Min Woo Lee, Jaeho Kang, Wonsang Chu, Eun Jin Park, Dong Oh Kang, Cheol Ung Choi, Suhng Wook Kim, Myung Ho Jeong, Soohyung Park, The Korea Acute Myocardial Infraction Registry study group, Seung-Woon Rha, Tae Hoon Ahn, Junghan Yoon, Hyo-Soo Kim, Ki-Bae Seung, Hyeon-Cheol Gwon, Shung Chull Chae, Chong-Jin Kim, Kwang Soo Cha, Jung-Hee Lee, Jei Keon Chae, Seung-Jae Joo, Chang-Hwan Yoon, Seung-Ho Hur, In-Whan Seong, Kyung-Kuk Hwang, Doo-Il Kim, Seok Kyu Oh, Jin-Yong Hwang, Myung Ho Jeong

**Affiliations:** 1 BK21 Graduate Program, Department of Biomedical Sciences, Korea University College of Medicine, Seoul, 02841 Republic of Korea; 2 Department of Cardiology, Cardiovascular Center, Guro Hospital, Korea University College of Medicine, Seoul, 08308 Republic of Korea; 3 Cardiovascular Research Institution, Korea University College of Medicine, Seoul, 02841 Republic of Korea; 4 Transdisciplinary Major in Learning Health Systems, Department of Healthcare Sciences, Graduate School, Seoul, 02841 Republic of Korea; 5 Research Institute of Health Science, Korea University, Seoul, 02841 Republic of Korea; 6 School of Health and Environmental Science, College of Health Sciences, Korea University, Seoul, 02841 Republic of Korea; 7 Department of Cardiology, Cardiovascular Center, Chonnam National University Hospital, Gwangju, 61469 Republic of Korea; 8 Korea University Guro Hospital, Seoul, Republic of Korea; 9 Gachon University Gil Medical Center, Incheon, Republic of Korea; 10 Wonju Severance Christian Hospital, Wonju, Republic of Korea; 11 Seoul National University Hospital, Seoul, Republic of Korea; 12 Seoul St. Mary’s Hospital, Seoul, Republic of Korea; 13 Samsung Medical Center, Seoul, Republic of Korea; 14 Kyungpook National University Hospital, Daegu, Republic of Korea; 15 Kyung Hee University Hospital at Gangdong, Seoul, Republic of Korea; 16 Pusan National University Hospital, Busan, Republic of Korea; 17 Yeungnam University Medical Center, Daegu, Republic of Korea; 18 Jeonbuk National University Hospital, Jeonju, Republic of Korea; 19 Jeju National University Hospital, Jeju, Republic of Korea; 20 Seoul National University Bundang Hospital, Seongnam, Republic of Korea; 21 Keimyung University Dongsan Medical Center, Daegu, Republic of Korea; 22 Chungnam National University Hospital, Daejeon, Republic of Korea; 23 Chungbuk National University Hospital, Cheongju, Republic of Korea; 24 Inje University Haeundae Paik Hospital, Busan, Republic of Korea; 25 Wonkwang University Hospital, Iksan, Republic of Korea; 26 Gyeongsang National University Hospital, Jinju, Republic of Korea; 27 Chonnam National University Hospital, Gwangju, Republic of Korea

**Keywords:** Environmental impact, Cardiology

## Abstract

Previous studies have reported the association between myocardial infarction (MI) and air pollution (AP). However, limited information is available regarding the long-term effects of AP on the relative incidence rates of ST-elevation MI (STEMI) and Non-ST-elevation MI (NSTEMI). We investigated the association between long-term exposure to AP and the incidence of STEMI. Between January 2006 and December 2015, a total of 45,619 eligible patients with Acute Myocardial Infarction (AMI) were enrolled in the Korea Acute MI Registry (KAMIR) and KAMIR-National Institutes of Health. Mixed-effect regression models were used to examine the association between the annual average ambient AP before MI onset and the incidence of STEMI, and to evaluate the association of AP with the incidence of in-hospital cardiogenic shock. After mixed-effect regression model analysis, particulate matter (PM) 10 µm or less in diameter (PM_10_) was associated with increased incidence of STEMI compared with NSTEMI (odds ratio [OR] 1.009, 95% Confidence Interval [CI] 1.002–1.016; *p* = 0.012). For in-hospital cardiogenic shock complication, PM_10_ and SO_2_ were associated with increased risk, PM_10_ (OR 1.033, 95% CI 1.018–1.050; *p* < 0.001), SO_2_ (OR 1.104, 95% CI 1.006–1.212; *p* = 0.037), respectively. Policy-level strategies and clinical efforts to reduce AP exposure are necessary to prevent the incidence of STEMI and severe cardiovascular complications.

## Introduction

Ischemic heart diseases (IHD), including acute myocardial infarction (AMI), constitute a significant global public health burden, being a leading cause of mortality and morbidity worldwide. AMI, in particular, is a major contributor to mortality in the Asia–Pacific region^1–4^. Patients with coronary artery disease (CAD) may experience complications related to air pollution (AP), such as increased hospitalization, re-admission, and early mortality^5–7^. Exposure to highly polluted air is one of the environmental factors that triggers AMI^8^. While both short and long-term effects of AP exposure have been investigated, the long-term consequences appear to outweigh the cumulative impact of short-term exposure^9^. Most studies have primarily focused on examining the association between short-term AP exposure and AMI^8,10,11^. However, only a few studies have reported on long-term AP exposure and compared the relative incidence of ST-elevation myocardial infarction (STEMI) and Non-ST-elevation myocardial infarction (NSTEMI). Particularly, the incidence of cardiogenic shock—a critical complication predominantly associated with STEMI—within the context of long-term AP exposure, has not been integrally investigated. Our previous studies demonstrated that AP exposure was associated with overall adverse clinical outcomes, including mortality, in AMI patients, considering both short and long-term exposure durations and follow-up periods^12,13^. As an extension of our prior findings, this study aims to further investigate the association between long-term average AP concentration and the relative risk of developing STEMI compared to a NSTEMI. Additionally, we aim to clarify the relationship between annual average AP concentration and the occurrence of cardiogenic shock, a complication observed to be more prevalent in STEMI patients.

## Methods

### Study protocols and population

**Figure 1:**
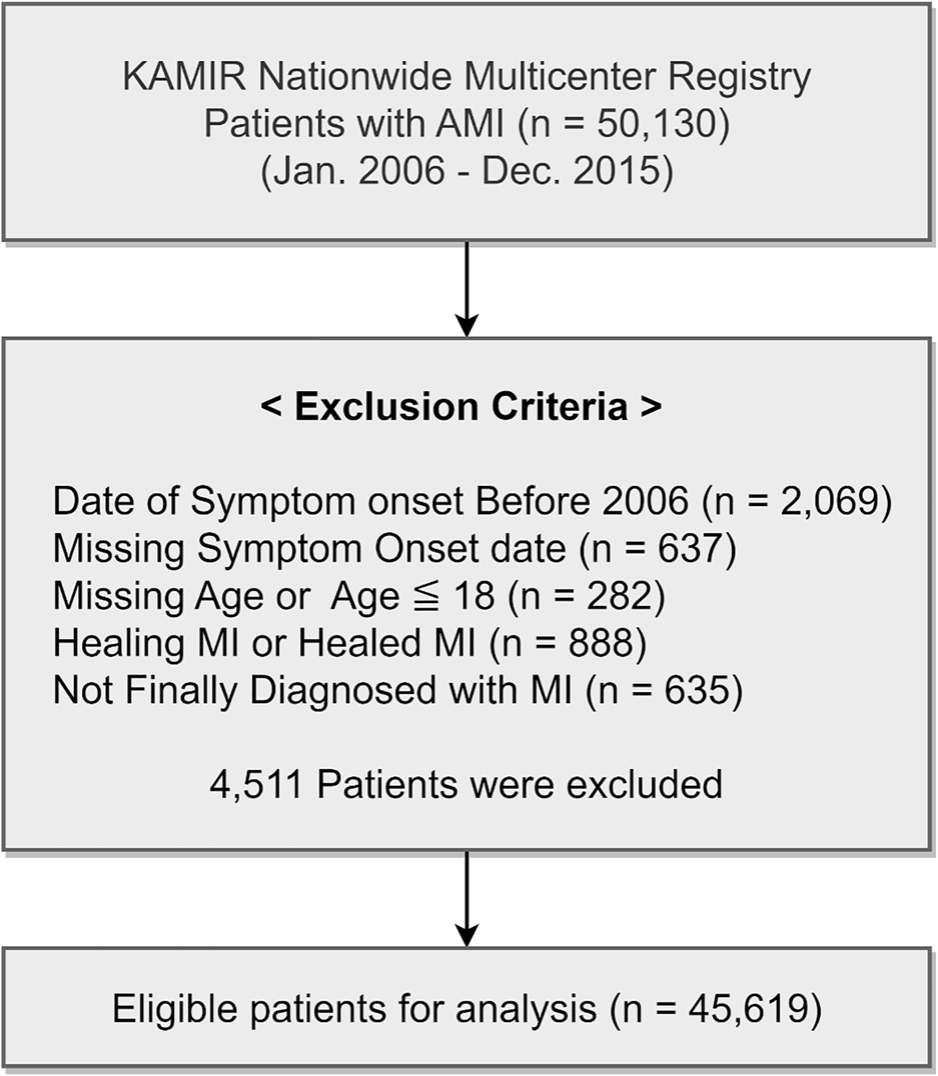
Study flow chart of patient enrollment. AMI = acute myocardial infarction; KAMIR = Korea Acute Myocardial Infarction Registry; MI = myocardial infarction.

The study subjects were enrolled in the Korea AMI registry (KAMIR) and KAMIR-National Institutes of Health (NIH). The KAMIR study protocol has been introduced previously^14^. KAMIR and KAMIR-NIH are nationwide prospective multicenter registration study series that aim to establish treatment guidelines and derive risk factors through the analysis of various clinical characteristics and follow-up of Korean AMI patients since October 2005 onwards. A flowchart of the study is shown in Fig. 1. A total of 50,130 patients with AMI were enrolled in the KAMIR and KAMIR-NIH between January 2006 and December 2015. The exclusion criteria were as follows: (1) date of symptom onset before 2006, (2) missing date of symptom onset, (3) age < 18 years, and (4) no final diagnosis of myocardial infarction (MI) at discharge.

### Ethical approval

This study was approved by the Institutional Review Board (IRB) of Korea University Guro Hospital (KUGH, #2016GR0740) and was conducted in accordance with the principles of the Declaration of Helsinki. Prior to giving written consent to participate, the participants or their legal guardians received a thorough and detailed explanation of the study procedures, both in written and verbal form.

### AP measurement

Hourly AP concentrations were provided by the Korean Ministry of Environment (http://www.airkorea.or.kr). In 2001, 329 monitoring stations nationwide began measuring the concentration of air pollutants. Measurement of air pollutants involved the β-ray absorption method for particulate matter (PM) 10 µm or less in diameter (PM_10_), the non-dispersive infrared method for carbon monoxide (CO), the pulse ultraviolet fluorescence method for sulfur dioxide (SO_2_), the chemiluminescence method for nitrogen dioxide (NO_2_), and the ultraviolet photometric method for ozone (O_3_). The concentration measurement of PM 2.5 µm or less in diameter (PM_2.5_) began in January 2015; therefore, annual average concentration values were not available during the patient enrollment period (2006–2015) and was excluded.

We transformed collected data into the daily average value, and then, the annual average value of air pollutants before the symptom day was calculated the way previous research was performed^13^. Each monitoring station was matched by the closest distance in a straight line to 68 hospitals registered in KAMIR to measure individual exposure concentration of air pollutants. Monitoring stations were selected based on hospital admission addresses for the following reasons: (1) Patient addresses were not included in the multicenter registry data. (2) As AMI is an emergency, it is assumed that the patient was admitted to an emergency room close to the workplace and residence at the time of symptom onset. If a pollutant measurement was missed due to a connection error with a monitoring station, the measurement of the next-nearest monitoring station was assigned. Symptom date was defined as the first occurrence of MI-related symptoms such as chest pain or dyspnea.

### Study definitions

The diagnosis of AMI was defined as an elevation in cardiac biomarkers (creatinine kinase-MB, and troponin I, or T) with typical changes on 12 leads electrocardiogram (ECG) or clinical symptoms. STEMI was diagnosed as a new ST-elevation segment measuring ≥ 1 mm from ≥ 2 contiguous leads on ECG. Patients with positive cardiac biomarkers but without ECG findings of STEMI were defined as NSTEMI. Cardiogenic shock was defined as a systolic blood pressure < 90 mmHg for > 30 min, the need for supportive management to maintain systolic blood pressure > 90 mmHg, and clinical signs of pulmonary congestion. A complication of cardiogenic shock is defined as its new onset after admission.

Individual cardiovascular risk factors, including hypertension (HTN), dyslipidemia (DL), diabetes mellitus (DM), prior cardiovascular disease, heart failure (HF), prior cerebrovascular disease (CVA), family history of CAD, and smoking history, were based on self reports by the patient.

### Statistical analysis

All statistical analyses were performed using R version 4.1.2. (R Core Team, 2021; R: Language and Environment for Statistical Computing; R Foundation for Statistical Computing, Vienna, Austria, URL: https://www.R-project.org/).

We compared the clinical and angiographic characteristics using a X^2^ test or Fisher’s exact test for categorical variables and Student’s t-test or Mann–Whitney rank test for continuous variables. In our analysis, X^2^ tests were used for categorical variables with expected cell frequencies of five or more; otherwise, Fisher’s exact test was applied. Continuous variables were analyzed with Student’s t-test if data were normally distributed (assessed by the Kolmogorov–Smirnov test) and with the Mann–Whitney rank test for non-normal distributions. Categorical data were expressed as percentages, and continuous variables were described as mean ± standard deviation.

We used generalized logistic mixed effect models with a random effect term for hospitals to examine the associations of each air pollutant with the incidence rate of STEMI and cardiogenic shock complication rates, and to account for hospital and regional effects such as accessibility and treatment plans. All variables used in the models for the incidence of STEMI and cardiogenic shock complications are presented in Table 1.

**Table 1:** Variables used in mixed-effects logistic regression models.

Response variable	Fixed effects	Random effect
${\text{log}}\left( {\frac{{{\text{P}}STEMI}}{{{\text{P}}NSTEMI}}} \right)$	Age, Sex, Body Mass Index, Smoking Status, Hypertension, Diabetes Mellitus, Dyslipidemia, Stroke, Heart Failure, Previous Ischemic Heart Disease, Family History of Coronary Artery Disease, Air Pollutant	Hospital
${\text{log}}\left( {\frac{{{\text{PCardiogenic}}\;{\text{Shock}}}}{{1 - {\text{PCardiogenic}}\;{\text{Shock}}}}} \right)$	Age, Sex, Body Mass Index, Smoking Status, Hypertension, Diabetes Mellitus, Dyslipidemia, Stroke, Heart Failure, Previous Ischemic Heart Disease, Family History of Coronary Artery Disease, STEMI Status, Percutaneous Coronary Intervention, Left Ventricular Ejection Fraction, Air Pollutant	Hospital

Using a multivariable model, we adjusted for potential confounding factors for STEMI incidence, including age, sex, body mass index (BMI), smoking status, HTN, DM, DL, stroke, HF, previous IHD, and family history of CAD. To analyze the incidence of cardiogenic shock complications, we considered the factors previously mentioned, in addition to STEMI status, percutaneous coronary intervention (PCI), and left ventricular ejection fraction (LVEF).

To assess and mitigate the risk of collinearity, we conducted correlation analyses and variance inflation factor (VIF) assessments among the included air pollutants. The VIF values obtained were below the commonly used threshold of 4, indicating that collinearity was unlikely to significantly impact the results of our regression analyses.

In the subgroup analysis, we conducted several stratified analyses using interaction terms for each specified group. For the STEMI group analysis, we included the following terms respectively: age, sex, HTN, DM, DL, CVA, HF, prior IHD, smoking, family history of CAD. In the cardiogenic shock group, STEMI status, PCI, and LVEF were added. The results were presented as adjusted odds ratios (OR) for logistic regression with corresponding 95% confidence intervals (CI). Statistical significance was defined as a *p*-value < 0.05.

## Results

A total of 45,619 patients with AMI were enrolled in our study. Of these, 20,526 were patients with NSTEMI and 25,093 were patients with STEMI. In our study population, compared with patients with NSTEMI, patients with STEMI were younger, male, had a higher smoking status, and had fewer underlying chronic diseases, such as DM, HTN, and DL. Moreover, patients with STEMI had more Killip class IV and a low LVEF and those were less likely to have a history of cardiovascular diseases, such as HF, CVA, and previous IHD. Among the angiographic parameters, the STEMI group had more PCI as the initial treatment for MI, lower multivessel coronary artery disease (MVD), and the left main artery as the culprit lesion. The cardiogenic shock complication rate during the index hospitalization was significantly higher in the STEMI group (Table 2).

**Table 2:** Baseline characteristics.

Variables	Total	NSTEMI (n = 20,526)	STEMI (n = 25,093)	*P* value
Age (year)	63.82 ± 12.77	65.10 ± 12.40	62.77 ± 12.97	< 0.001
Sex (male)	32,921 (72.2)	14,042 (68.4)	18,879 (75.2)	< 0.001
Body mass index (kg/m^2^)	23.98 ± 3.25	23.94 ± 3.27	24.01 ± 3.23	0.036
Systolic blood pressure (mmHg)	129.69 ± 28.25	133.71 ± 27.38	126.37 ± 28.53	< 0.001
Diastolic blood pressure (mmHg)	78.89 ± 16.66	80.18 ± 15.87	77.82 ± 17.22	< 0.001
Heart rate (bpm)	78.19 ± 19.90	79.70 ± 19.36	76.96 ± 20.25	< 0.001
Left ventricular ejection fraction (%)	51.96 ± 11.86	53.51 ± 12.20	50.66 ± 11.41	< 0.001
Killip Class 4	2389 (5.2)	552 (2.7)	1837 (7.3)	< 0.001
Hypertension	22,776 (49.9)	11,203 (54.6)	11,573 (46.1)	< 0.001
Diabetes mellitus	12,596 (27.6)	6418 (31.3)	6178 (24.6)	< 0.001
Dyslipidemia	5082 (11.1)	2571 (12.5)	2511 (10.0)	< 0.001
Cerebrovascular disease	3030 (6.6)	1659 (8.1)	1371 (5.5)	< 0.001
Heart failure	842 (1.8)	582 (2.8)	260 (1.0)	< 0.001
Smoking history	26,337 (57.7)	11,077 (54.0)	15,260 (60.8)	< 0.001
Current smoker	18,853 (41.3)	7276 (35.4)	11,577 (46.1)	< 0.001
Family history of heart disease	3285 (7.2)	1501 (7.3)	1784 (7.1)	0.404
Previous ischemic heart disease	7027 (15.4)	4150 (20.2)	2877 (11.5)	< 0.001
Previous PCI	3346 (7.3)	1968 (9.6)	1378 (5.5)	< 0.001
Previous MI	2164 (4.7)	1265 (6.2)	899 (3.6)	< 0.001
Previous CABG	371 (0.8)	268 (1.3)	103 (0.4)	< 0.001
Previous angina	2793 (6.1)	1733 (8.4)	1060 (4.2)	< 0.001
Initial treatment of MI				
Thrombolysis	990 (2.2)	0 (0.0)	990 (3.9)	< 0.001
CABG	933 (2.0)	556 (2.7)	377 (1.5)	< 0.001
PCI	39,847 (87.3)	16,329 (79.6)	23,518 (93.7)	< 0.001
Multi-vessel disease	21,542 (47.2)	9942 (48.4)	11,600 (46.2)	< 0.001
Left main disease	1661 (3.6)	957 (4.7)	704 (2.8)	< 0.001
Infarct-related artery				
Left main	886 (1.9)	522 (2.5)	364 (1.5)	< 0.001
Left anterior descending artery	18,963 (41.6)	6987 (34.0)	11,976 (47.7)	< 0.001
Left circumflex artery	6849 (15.0)	4596 (22.4)	2253 (9.0)	< 0.001
Right coronary artery	13,489 (29.6)	4719 (23.0)	8770 (34.9)	< 0.001
Cardiogenic Shock Complications	2317 (5.1)	554 (2.7)	1763 (7.0)	< 0.001

Data is expressed as number (percentage), mean ± standard deviation. CABG = coronary artery bypass grafting, NSTEMI = non-ST-elevation myocardial infarction, PCI = percutaneous coronary intervention, MI = myocardial infarction, STEMI = ST-elevation myocardial infarction.

In Table 3, we observed the median value of annual average concentrations was 0.049 part per million (ppm) for SO_2_, 0.6088 ppm for CO, 0.0211 ppm for O_3_, 0.0259 ppm for NO_2_, and 50.53 μg/m^3^ for PM_10_.

**Table 3:** Distribution for annual average air pollution concentration before symptom date.

	SO_2_ (ppm)	CO (ppm)	O_3_ (ppm)	NO_2_ (ppm)	PM_10_ (µg/m^3^)
Min	0.0014	0.2028	0.0037	0.0070	18.25
Q1	0.0041	0.5312	0.0176	0.0218	44.65
Median	0.0049	0.6088	0.0211	0.0259	50.53
Q3	0.0061	0.7211	0.0244	0.0342	58.67
Max	0.0136	1.4976	0.0437	0.0812	99.14
IQR	0.0020	0.1899	0.0068	0.0124	14.02
Mean	0.0053	0.6276	0.0210	0.0284	52.57

CO = carbon monoxide, IQR = interquartile range, NO_2_ = nitrogen dioxide, O_3_ = ozone, PM_10_ = particulate matter 10 µm or less in diameter, ppm = part per million, Q1 = 1st quintile, Q3 = 3rd quintile, SO_2_ = sulfur dioxide.

**Figure 2:**
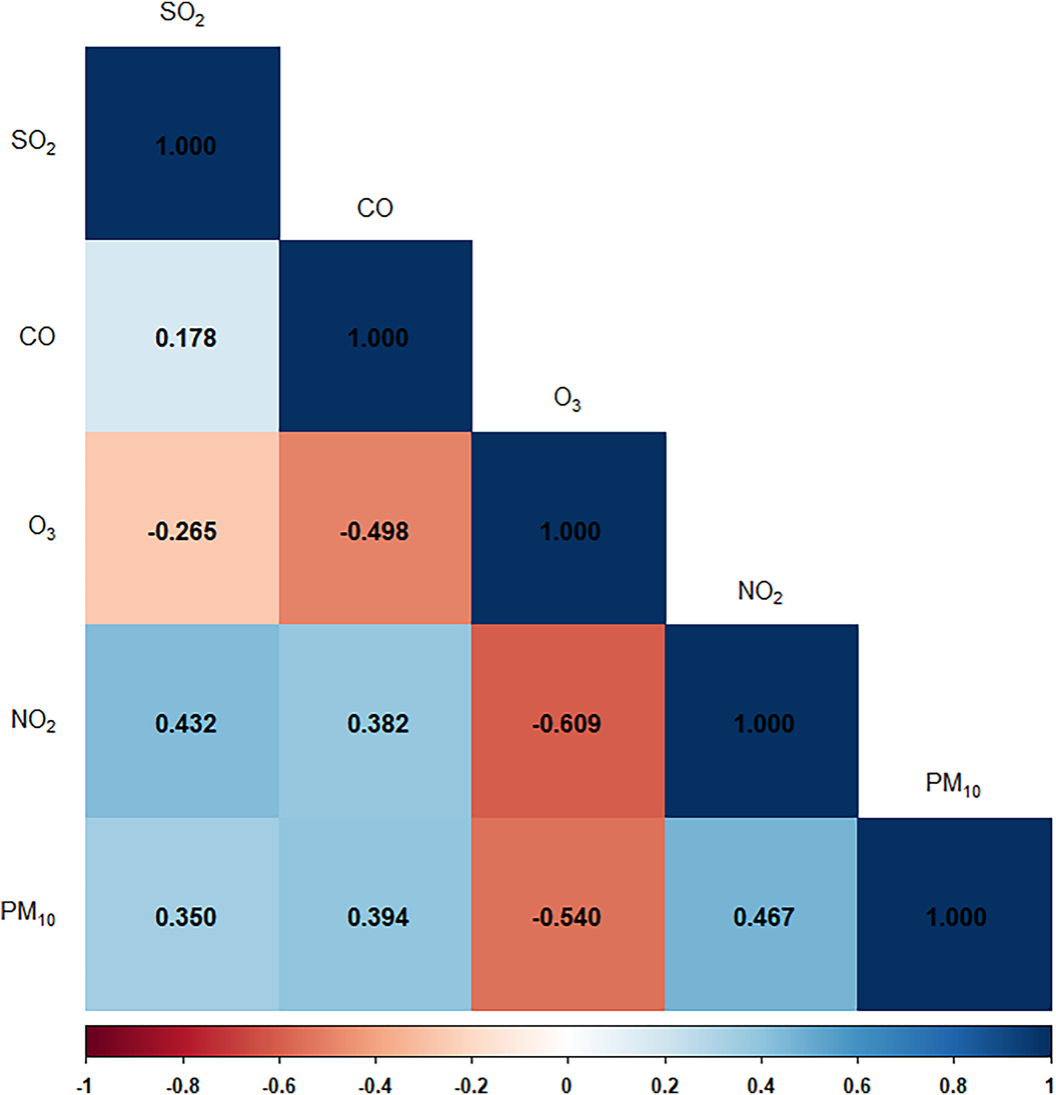
Spearman correlation coefficients for annual average concentrations of air pollutants. CO = carbon monoxide; NO_2_ = nitrogen dioxide; PM_10_ = particulate matter 10 µm or less in diameter.

In the Spearman rank correlation analysis using the average annual concentrations after the symptom date, most air pollutants showed a positive correlation (r = 0.178–0.467); however, O_3,_ and other air pollutants, showed a negative correlation (r = − 0.265 to − 0.609; Fig. 2).

After mixed-effect regression model analysis, no difference was observed for most air pollutants except PM_10_, which was associated with increased incidence of STEMI compared with NSTEMI for each 1 μg/m^3^ increase (OR 1.009, 95% CI 1.002–1.016; *p* = 0.012; Table 4). For in-hospital cardiogenic shock complication, each 1 μg/m^3^ increase of PM_10_ and each 1 part per billion (ppb) increase of SO_2_ were associated with increased risk: PM_10_ (OR 1.033, 95% CI 1.018–1.050; *p* < 0.001), SO_2_ (OR 1.104, 95% CI 1.006–1.212; *p* = 0.037), respectively. In contrast, for each 1 ppb increase in O_3_ was negatively correlated with cardiogenic shock (OR 0.891; 95% CI 0.857–0.928; *p* < 0.001; Table 5).

**Table 4:** Univariate and multivariate regression analysis of the incidence of STEMI compared with NSTEMI regarding annual average concentration of each air pollutant before symptom date.

	Univariate		Multivariate	
	OR (95% CI)	*P* value	OR (95% CI)	*P* value
SO_2_ (ppb)	1.044 (0.998–1.093)	0.060	1.041 (0.995–1.089)	0.084
CO (ppm)	1.037 (0.978–1.100)	0.225	1.036 (0.977–1.099)	0.236
O_3_ (ppb)	0.990 (0.972–1.009)	0.293	0.989 (0.971–1.007)	0.239
NO_2_ (ppb)	1.000 (0.987–1.012)	0.943	0.999 (0.987–1.012)	0.914
PM_10_ (µg/m^3^)	1.008 (1.001–1.014)	0.027	1.009 (1.002–1.016)	0.012

Adjusted for Age, body mass index, diabetes mellitus, dyslipidemia, heart failure, hypertension, previous ischemic heart disease, sex, smoking status, stroke, and family history of CAD. CI = confidence interval, CO = carbon monoxide, NO_2_ = nitrogen dioxide, O_3_ = ozone, OR = odds ratio, PM_10_ = particulate matter 10 µm or less in diameter, ppb = part per billion, ppm = part per million, SO_2_ = sulfur dioxide.

**Table 5:** Univariate and multivariate regression analysis between the incidence of cardiogenic shock events and the annual average concentration of each air pollutant before the symptom date of myocardial infarction.

	Univariate		Multivariate	
	OR (95% CI)	*P* value	OR (95% CI)	*P* value
SO_2_ (ppb)	1.122 (1.021–1.234)	0.017	1.104 (1.006–1.212)	0.037
CO (ppm)	1.090 (0.960–1.238)	0.182	1.076 (0.949–1.219)	0.254
O_3_ (ppb)	0.889 (0.853–0.926)	< 0.001	0.891 (0.857–0.928)	< 0.001
NO_2_ (ppb)	1.013 (0.985–1.041)	0.365	1.015 (0.988–1.042)	0.269
PM_10_ (µg/m^3^)	1.036 (1.019–1.053)	< 0.001	1.033 (1.018–1.050)	< 0.001

Adjusted for age, body mass index, diabetes mellitus, dyslipidemia, heart failure, hypertension, left ventricular ejection fraction, percutaneous coronary intervention, previous ischemic heart disease, sex, smoking status, STEMI status, stroke, and family history of CAD. CI = confidence interval, CO = carbon monoxide, NO_2_ = nitrogen dioxide, O_3_ = ozone, OR = odds ratio, PM_10_ = particulate matter 10 µm or less in diameter, ppb = part per billion, ppm = part per million, SO_2_ = sulfur dioxide.

When STEMI and each air pollutant were analyzed in subgroups, the results showed there was a significant association with a decrease in STEMI incidence for every 1 ppb increase of NO_2_ in CVA patients. In the absence of HTN, there was an increase in STEMI incidence for every 1 μg/m^3^ increase PM_10_ (Supplementary Figs. 1–5). In subgroup analyses used to evaluate the risk of cardiogenic shock with AP exposure, it is shown that there was a significant association between increasing cardiogenic shock complication rate and for each 1 ppb increase of NO_2_ in patients with no history of PCI, for each 1 μg/m^3^ increase of PM_10_ in patients with prior IHD or without PCI treatment, and for each 1 ppb increase in SO_2_ with HF patients or prior IHD (Supplementary Figs. 6–10).

## Discussion

In the population of AMI patients, we performed a large registry-based analysis to evaluate the association between long-term exposure to AP. In this study, using a nationwide prospective clinical registry, we found associations between elevated levels of AP and a higher incidence of STEMI relative to NSTEMI. Additionally, our findings suggest an association between increased AP concentrations and a heightened incidence of cardiogenic shock complications, which have been linked to increased overall mortality. The results of this study showed that long-term exposure to high levels of PM_10_ is associated with an increased risk of STEMI. Moreover, this study demonstrates that PM_10_ and SO_2_ may impact on the development of cardiogenic shock complication in patients with AMI.

Air pollutants comprise complex mixtures that are compounded with gases, including SO_2_, NO_2_, CO, O_3_, and PM, including PM_10_ and PM_2.5_^15^. Although it may intuitively seem that AP poses a health risk mostly in the form of respiratory disease, many epidemiological and clinical studies have suggested that the majority of the adverse effects of AP are associated with the cardiovascular system^16–18^. Previous studies demonstrated that AP exposure is associated with endothelial injury and inflammation, indicating that it can trigger cardiovascular events^19–21^. Moreover, recent study demonstrated that air pollution may induce plaque rupture and is associated with macrophage infiltrates in coronary plaques and it is well reported that plaque rupture portends a worse prognosis in MI patients^22^. By integrating these insights with the concept of the exposome—defined as the totality of environmental exposures—this discussion broadens to highlight the need to assess the cumulative impact of such exposures, particularly key inflammation drivers like air pollution, on cardiovascular risk and outcomes^23^.

Regarding the association between AP and CVD, many studies have demonstrated that short-term exposure to AP increases the incidence of an acute coronary syndrome (ACS)^24–26^. In contrast, the present study investigated the effects of long-term exposure to AP, which was the major novelty of our research. The number of studies on the long-term effects of AP exposure are increasing. The ESCAPE (European Study of Cohorts for Air Pollution Effects) study, the increase in PM_10_ and PM_2.5_ during the long-term follow-up period increases the risk of ACS^27^. However, these studies mainly focus on mortality or overall clinical outcome^17,27–29^. Researchers have rarely compared the risk of developing STEMI with that of NSTEMI. To the best of our knowledge, this is the first study to demonstrate a long-term association between AP exposure and the relative incidence of STEMI compared with that of NSTEMI in the Asia–Pacific region.

Our present study was shown that the risk of developing STEMI increased compared to NSTEMI according to the 1-year average PM_10_ concentration before symptom onset. These results indicate that STEMI contributes more than NSTEMI to an increased risk of MI according to the AP concentration. Studies on short-term exposure have reported that elevated AP exposure highly triggers the development of STEMI compared with that of NSTEMI^30,31^. However, some studies have also reported greater incidence of NSTEMI than that of STEMI due to AP exposure^32^. The inconsistent results can be attributed to differences in exposure periods, geographic location, pollutant concentration level, study population, and statistical methods used for analysis^8,10^.

In addition, this study is meaningful in that we clarified the effect of AP exposure on the risk of severe complications of cardiogenic shock. Our main findings showed that an increased AP concentration was associated with an increase incidence of cardiogenic shock complication. Cardiogenic shock occurs in approximately 5–13% of AMI patients^33^. Moreover, AMI itself was an important etiology contributing to 80% incidences of cardiogenic shock^34^. Cardiogenic shock is associated with poor prognosis for high rate of adverse events even with appropriate treatment, with an in-hospital mortality of 20–40% and a 1-year mortality rate of up to 50%^33^.

Although the pathophysiology of cardiogenic shock is not fully understood, it is known that the systemic inflammatory response, release of inflammatory cytokines, and increase in the concentration of nitric oxide (NO) are involved in inappropriate vasodilation after peripheral vascular constriction to compensate for the reduction in myocardial contractility^35,36^. Exposure to AP is related to oxidative stress and systemic inflammation^14,16–18^ and it adversely affects vascular homeostasis through the production of superoxide and the uncoupling of NO synthase^37^. These results add evidence for the development of cardiogenic shock and its subsequent poor prognosis.

In our previously published study, the 1-year average AP concentration before the onset of symptoms was associated with an increase in 30-day short-term mortality^13^. Studies using the same registry reported that STEMI patients exhibited not only higher short-term mortality but also an elevated incidence of cardiogenic shock compared to NSTEMI patients^38^. These results underline the critical need for a consolidated approach in research to further understand the mechanisms linking AP exposure, the differential impact on STEMI versus NSTEMI, and the subsequent risk of cardiogenic shock and cardiac death^33^.

This study strongly suggests that reducing exposure to high concentrations of AP is crucial for reducing the occurrence of potential MI and mortality. This holds true not only for the high-risk group but also for the low-risk group of AMI. It is necessary to reduce the occurrence of potential MI and mortality, even if STEMI appears to be relatively safe because of its younger age and lower co-morbidity rates than NSTEMI (Table 1). These efforts should be accompanied by policy strategies and clinical practice.

This study has several limitations. First, because of the limited sampling data available for PM_2.5_, the associations with clinical events may have been relatively low. PM_2.5_ data was only available for 2015, the final year of our study period, limiting longitudinal analysis. Evidence suggests that PM size is related to cardiovascular morbidity and mortality^29,38,39^. Further studies with new data are needed to evaluate the impact of PM_2.5_ in AMI patients in the future with our KAMIR data with later than 2015 registry database. Second, because the patients’ addresses were not available, the direct exposure level determined for the patients could be incorrect. It was assumed that patients were admitted to a nearby emergency room at the time of symptom onset. However, some patients who visited other local hospitals or were transferred may have been misclassified, requiring careful interpretation of the results. Third, because of the limitations of the study design, although confounding factors were adjusted for, the results for residential addresses and socioeconomic variables should be carefully considered. We aimed to assess adjusted adequate variables which could be potentially relevant factors using a multivariable model. Finally, although data used in this research were collected by the attending hospitals well-trained, multicenter registry is need to interpret with consideration for several characteristics such as a gap among each hospitals and data such as input errors and misclassification.

In conclusion, We observed that high concentration of air pollutants, particularly of PM_10_, which is an environmental risk factor was associated with an increased incidence of STEMI. Moreover, PM_10_ and SO_2_ levels were risk factors for in-hospital cardiogenic shock complication after MI. This study emphasizes on the need of developing a policy-level strategy and clinical efforts to reduce AP exposure and prevent the incidence of STEMI and severe cardiovascular complications.

### Supplementary Information

Supplementary Figures.

## Data Availability

Data and materials cannot be shared publicly because of the KAMIR group policy. Data are available from the Chonnam National University Hospital Institutional Data Access/Ethics Committee (contact via research manager) for researchers who meet the criteria for access to confidential data.
