# Supplementary material for: Long-term air pollution exposure is associated with higher incidence of ST-elevation myocardial infarction and in-hospital cardiogenic shock
Source: Sci Rep. 2024 Feb 29;14:4976. doi: 10.1038/s41598-024-55682-6 (PMC10904831; doi:10.1038/s41598-024-55682-6)
Supplement: Supplementary file 1 — Supplementary Figures. [file 41598_2024_55682_MOESM1_ESM.docx]

**Supplementary Figure 1.** **Subgroup analysis for the adjusted odds ratio and 95% confidence interval of the incidence of STEMI compared with NSTEMI according to an increase of 1 part per billion SO_2_ before the onset of symptoms.**


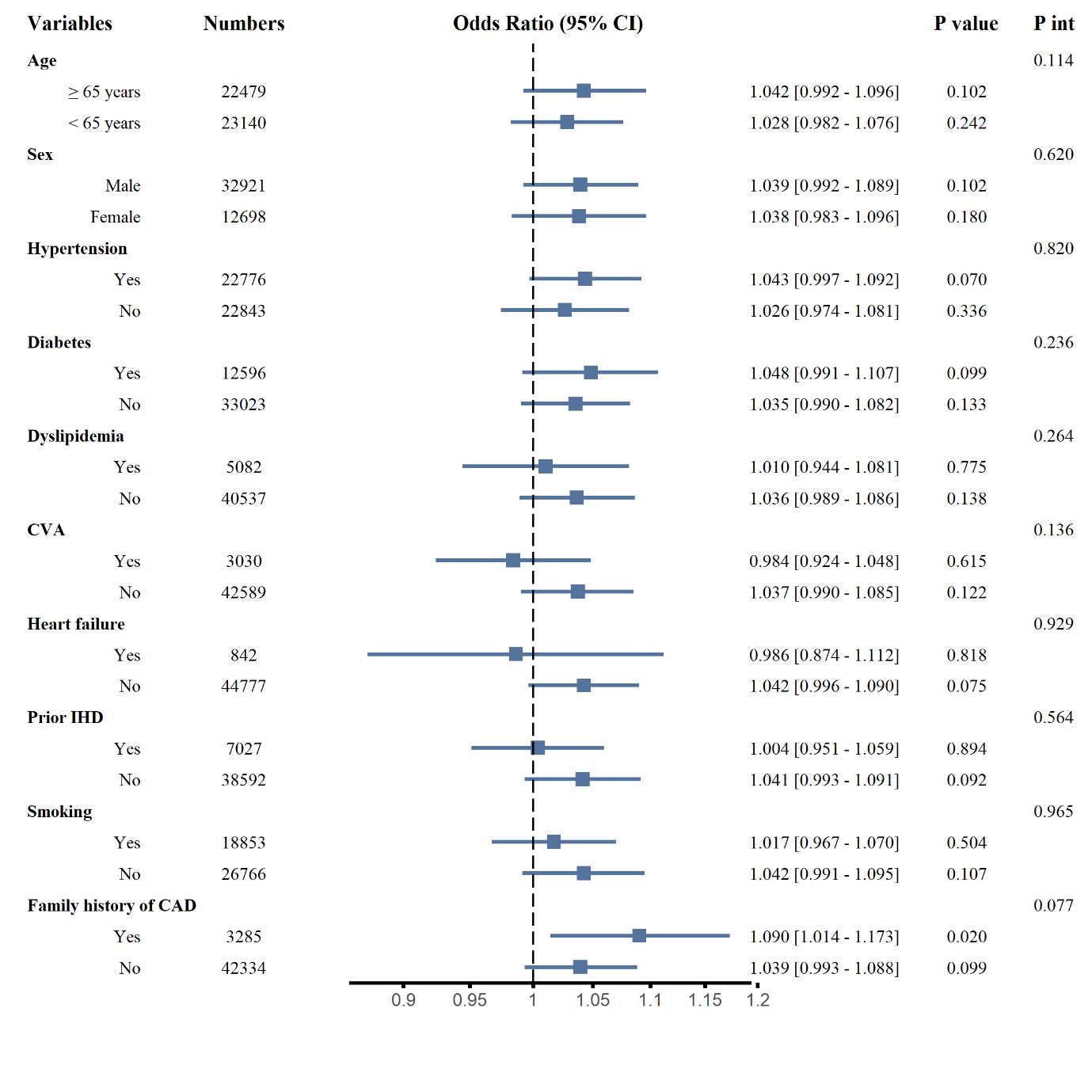


CI = confidence interval, CVA = cerebrovascular accident, IHD = ischemic heart disease, CAD = coronary artery disease.

**Supplementary Figure 2.** **Subgroup analysis for adjusted odds ratio and 95% confidence interval of the incidence of STEMI compared with NSTEMI according to an increase of 0.1 part per million CO.**


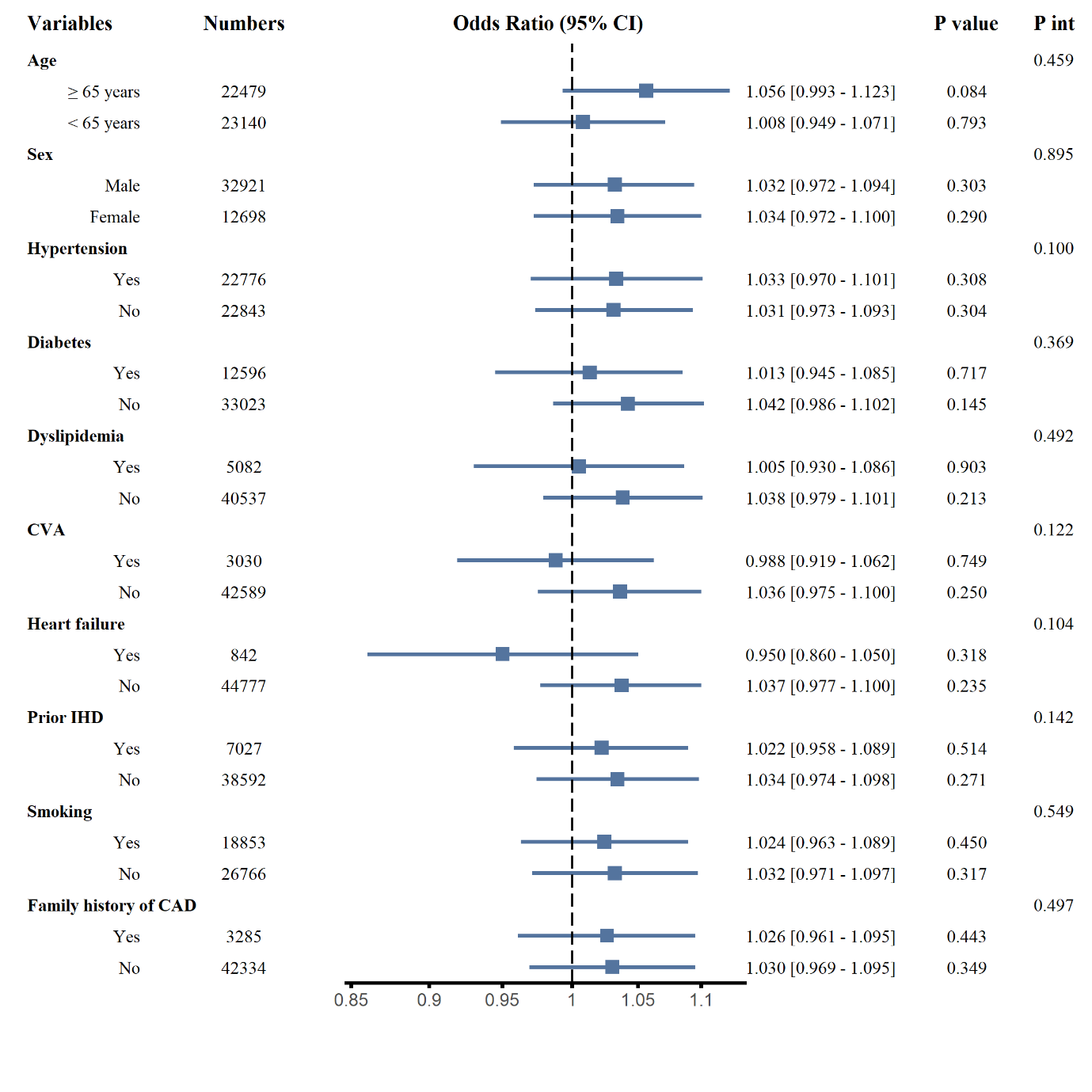
 CI = confidence interval, CVA = cerebrovascular accident, IHD = ischemic heart disease, CAD = coronary artery disease.

**Supplementary Figure 3.** **Subgroup analysis for the adjusted odds ratio and 95% confidence interval of the incidence of STEMI compared with NSTEMI according to an increase of 1 part per billion O_3_ before the onset of symptoms.**


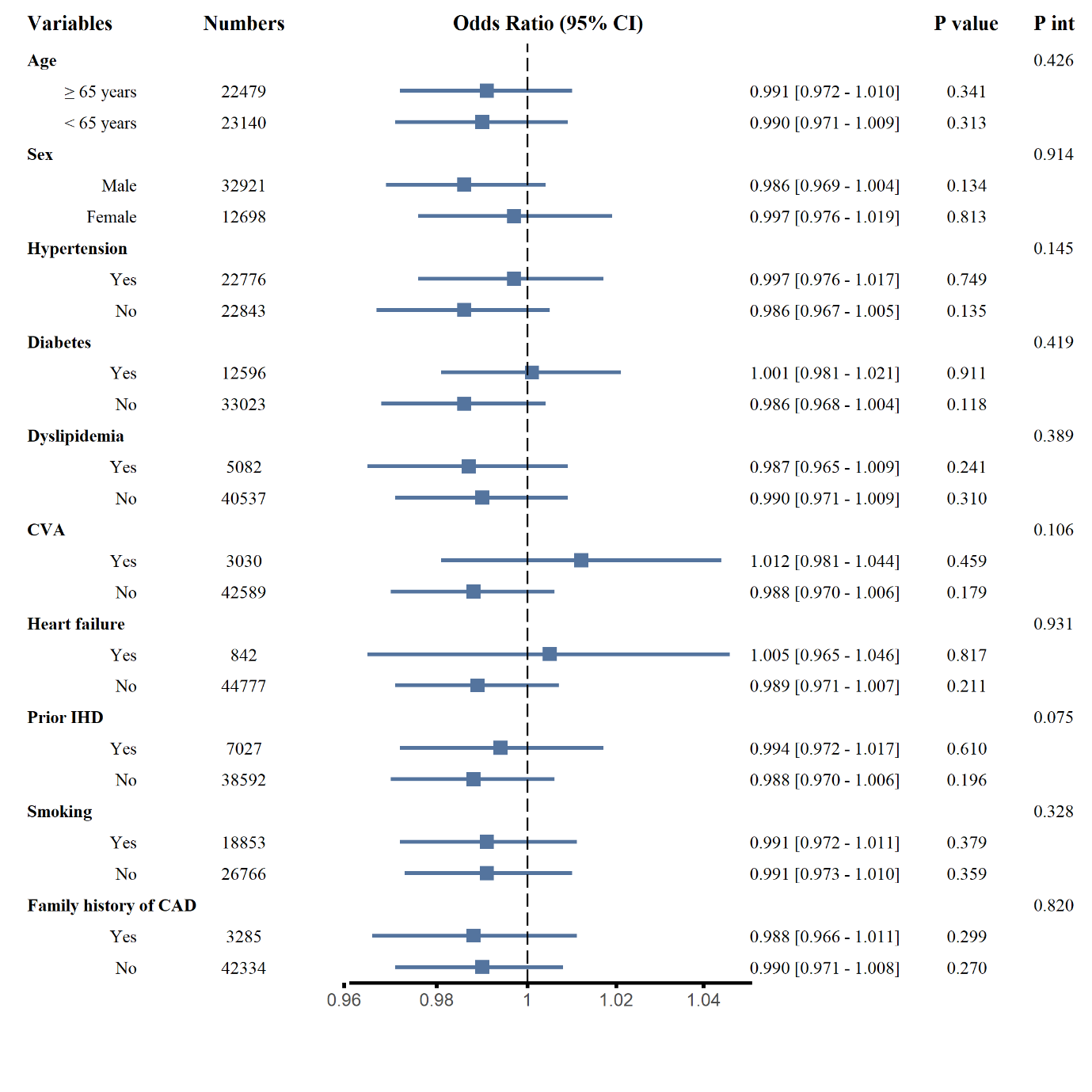
CI = confidence interval, CVA = cerebrovascular accident, IHD = ischemic heart disease, CAD = coronary artery disease.

**Supplementary Figure 4.** **Subgroup analysis for the adjusted odds ratio and 95% confidence interval of the incidence of STEMI compared with NSTEMI according to an increase of 1 part per billion NO_2_ before the onset of symptoms.**


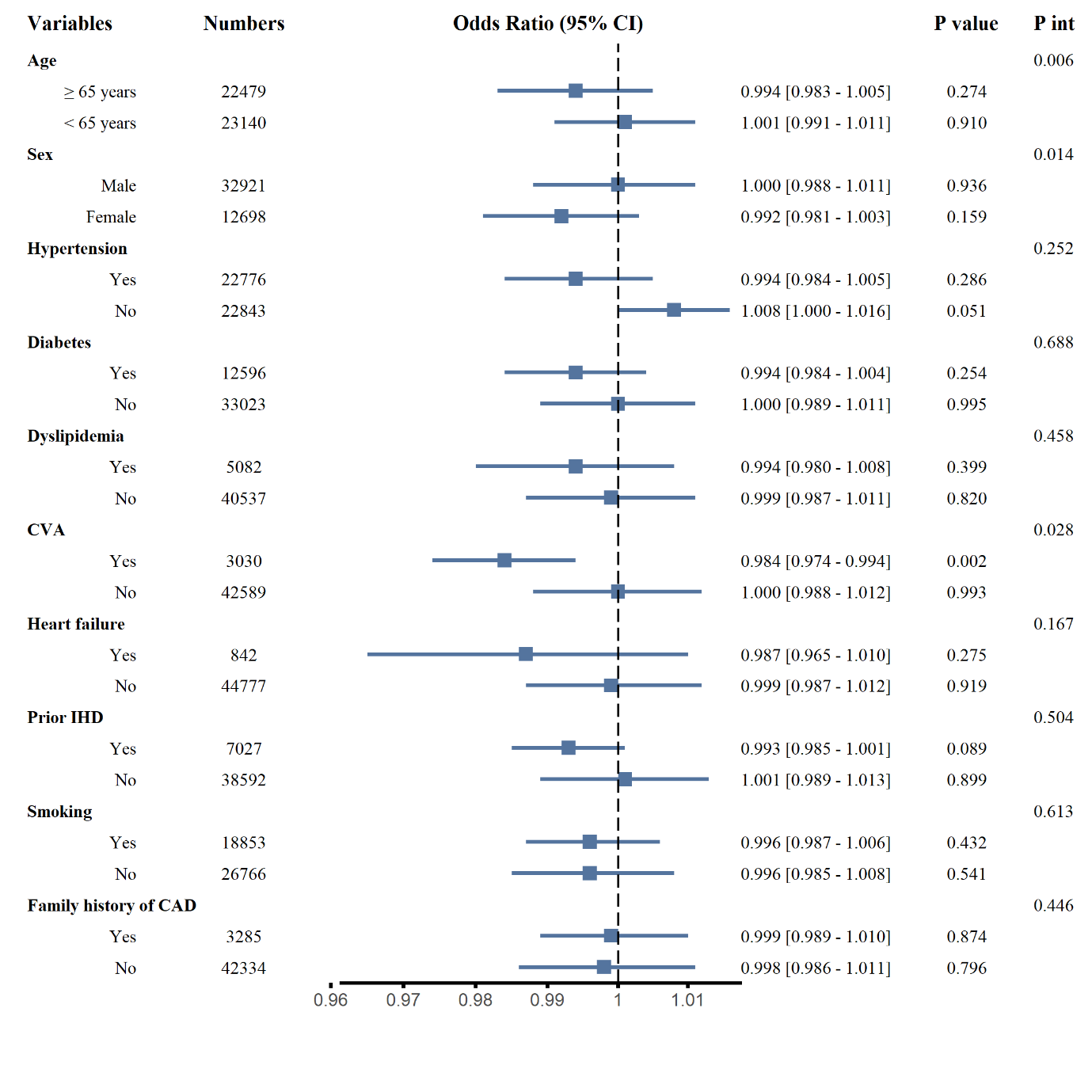
CI = confidence interval, CVA = cerebrovascular accident, IHD = ischemic heart disease, CAD = coronary artery disease.

**Supplementary Figure 5.** **Subgroup analysis for the adjusted odds ratio and 95% confidence interval of the incidence of STEMI compared with NSTEMI according to an increase of 1 µg/m^3^ PM_10_ before the symptom date.**


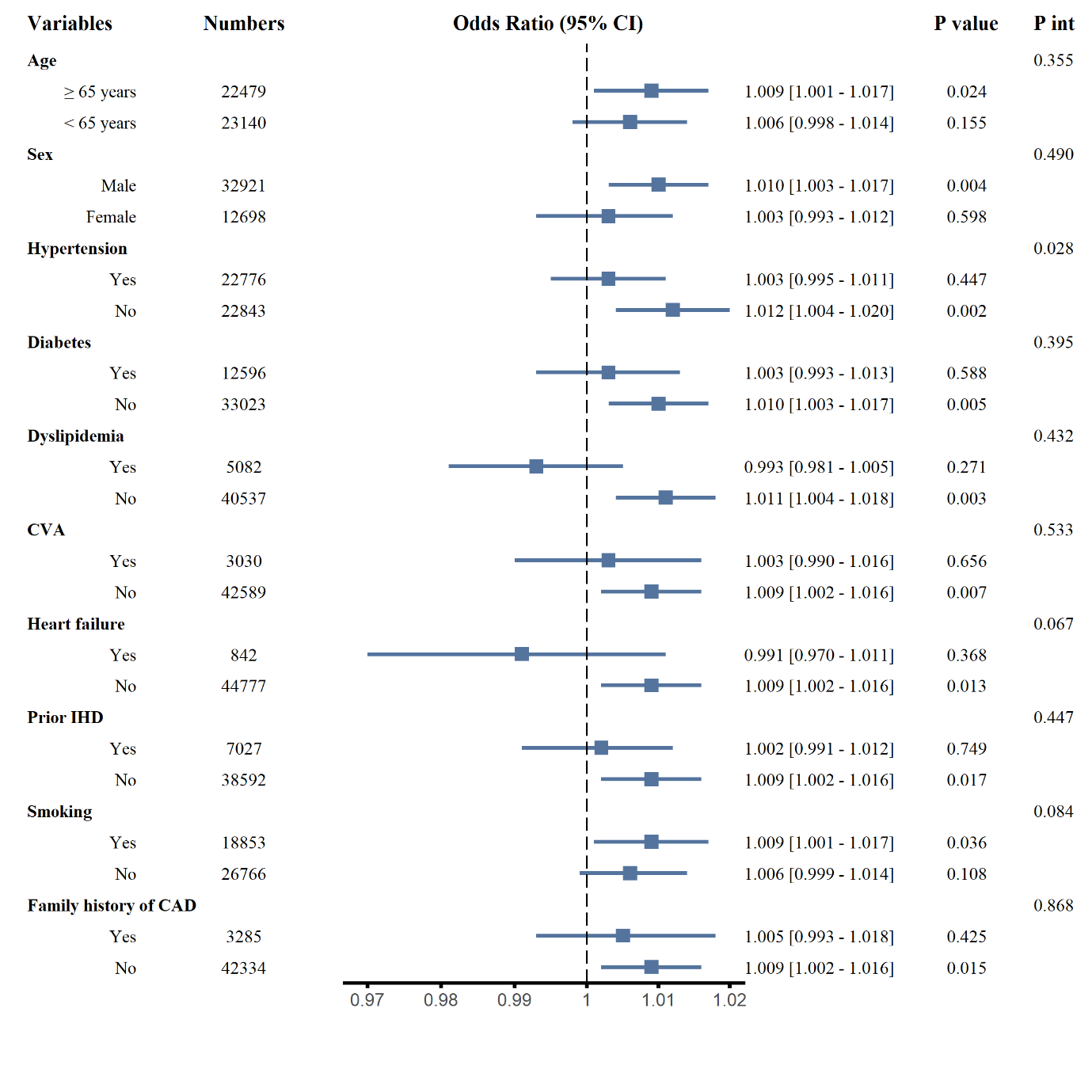
CI = confidence interval, CVA = cerebrovascular accident, IHD = ischemic heart disease, CAD = coronary artery disease.

**Supplementary Figure 6. Subgroup analysis for the adjusted odds ratio and 95% confidence interval of the incidence of in-hospital cardiogenic shock according to an increase of 1 part per billion SO_2_ before symptom onset.**


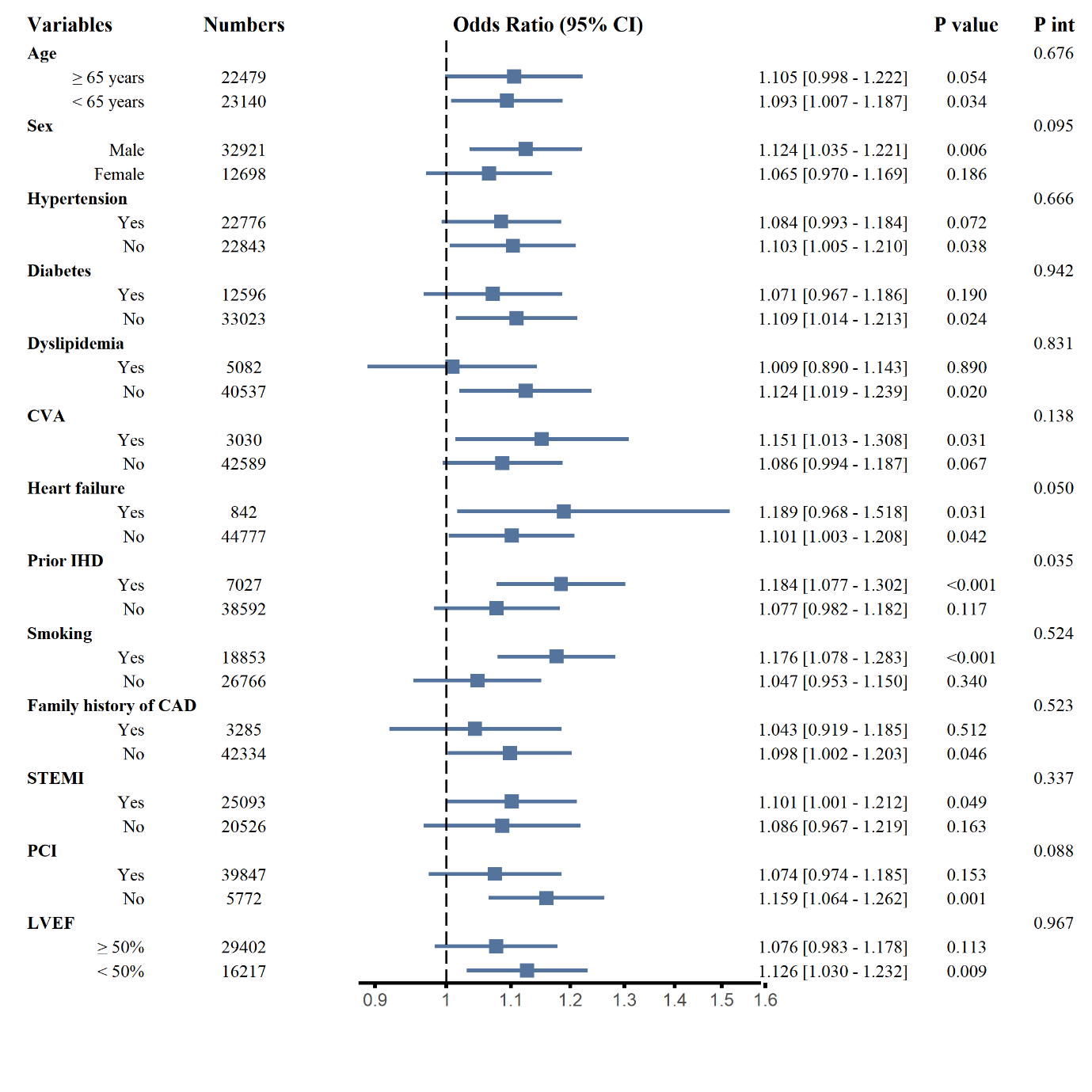
CI = confidence interval, CVA = cerebrovascular accident, IHD = ischemic heart disease, CAD = coronary artery disease, STEMI = ST-elevation myocardial infarction, PCI = percutaneous coronary intervention, LVEF = left ventricular ejection fraction.

**Supplementary Figure 7. Subgroup Analysis for adjusted odds ratio and 95% confidence interval of the incidence of in-hospital cardiogenic shock according to an increase of 0.1 part per million CO before symptom date.**


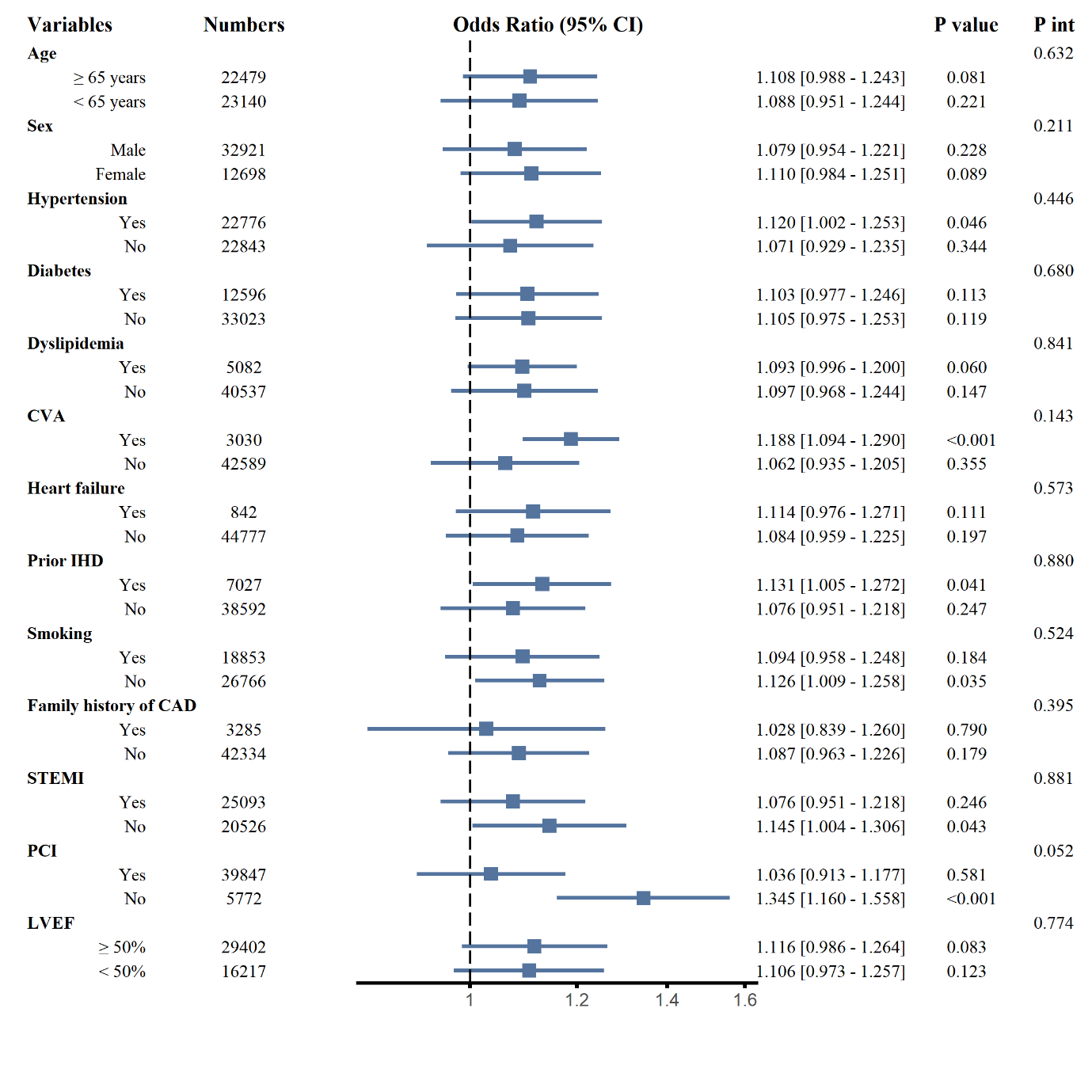


CI = confidence interval, CVA = cerebrovascular accident, IHD = ischemic heart disease, CAD = coronary artery disease, STEMI = ST-elevation myocardial infarction, PCI = percutaneous coronary intervention, LVEF = left ventricular ejection fraction.

**Supplementary Figure 8. Subgroup analysis for the adjusted odds ratio and 95% confidence interval of the incidence of in-hospital cardiogenic shock according to an increase of 1 part per billion O_3._**


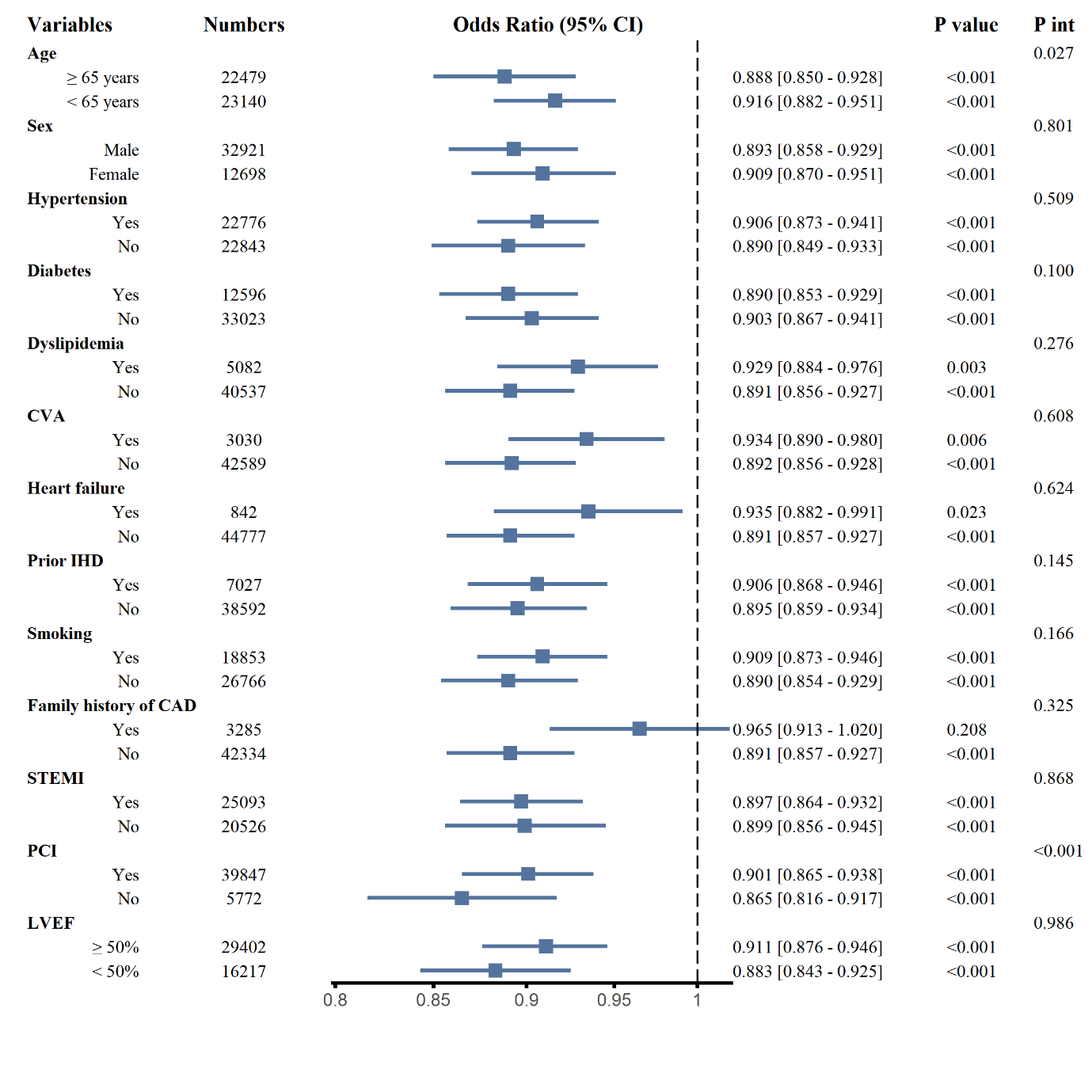
CI = confidence interval, CVA = cerebrovascular accident, IHD = ischemic heart disease, CAD = coronary artery disease, STEMI = ST-elevation myocardial infarction, PCI = percutaneous coronary intervention, LVEF = left ventricular ejection fraction.

**Supplementary Figure 9.** **Subgroup analysis for the adjusted odds ratio and 95% confidence interval of the incidence of in-hospital cardiogenic shock according to an increase of 1 part per billion NO_2._**


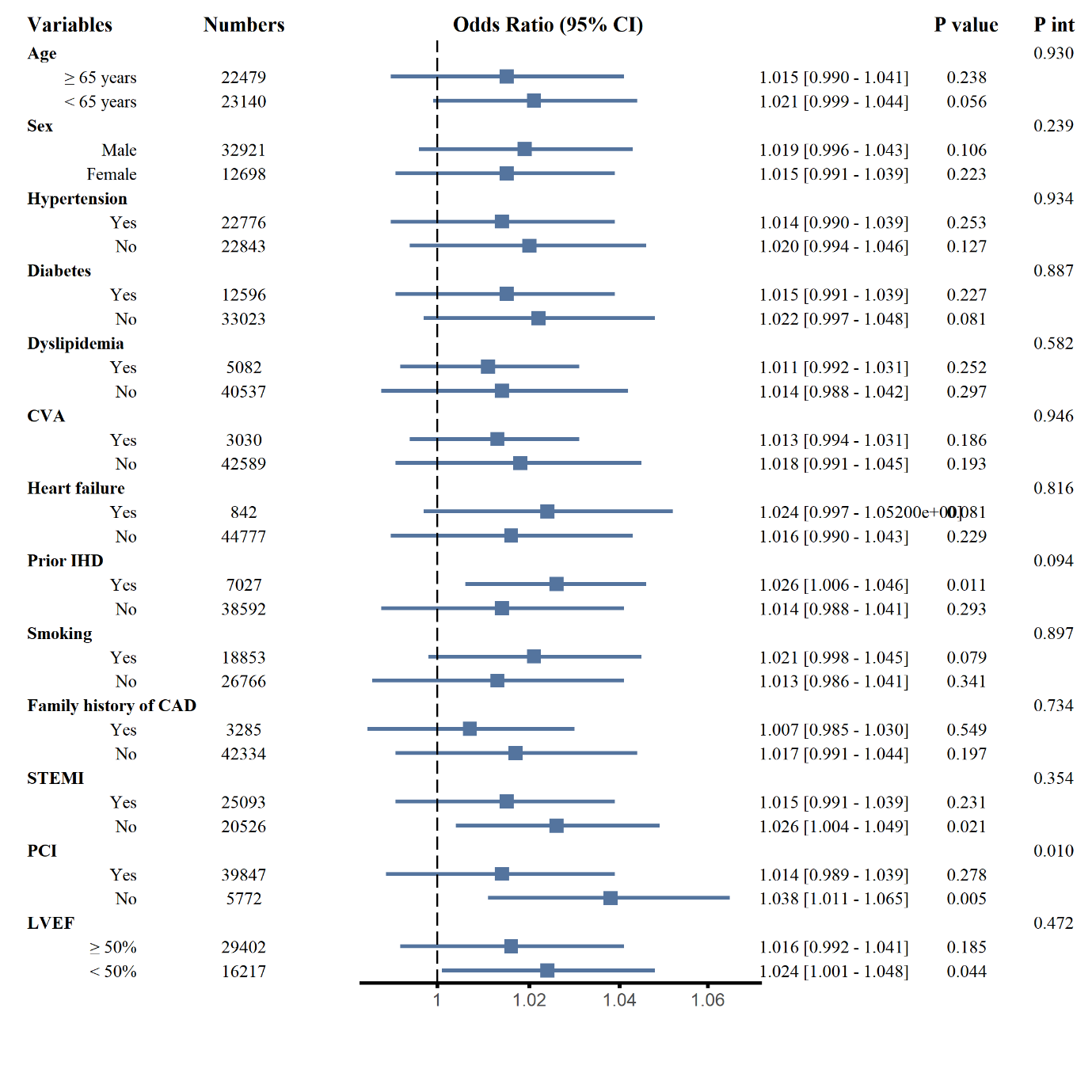
CI = confidence interval, CVA = cerebrovascular accident, IHD = ischemic heart disease, CAD = coronary artery disease, STEMI = ST-elevation myocardial infarction, PCI = percutaneous coronary intervention, LVEF = left ventricular ejection fraction.

**Supplementary Figure 10.** **Subgroup analysis for the adjusted odds ratio and 95% confidence interval of the incidence of in-hospital cardiogenic shock according to the increase 1 µg/m^3^ PM_10_ before the symptom date.**


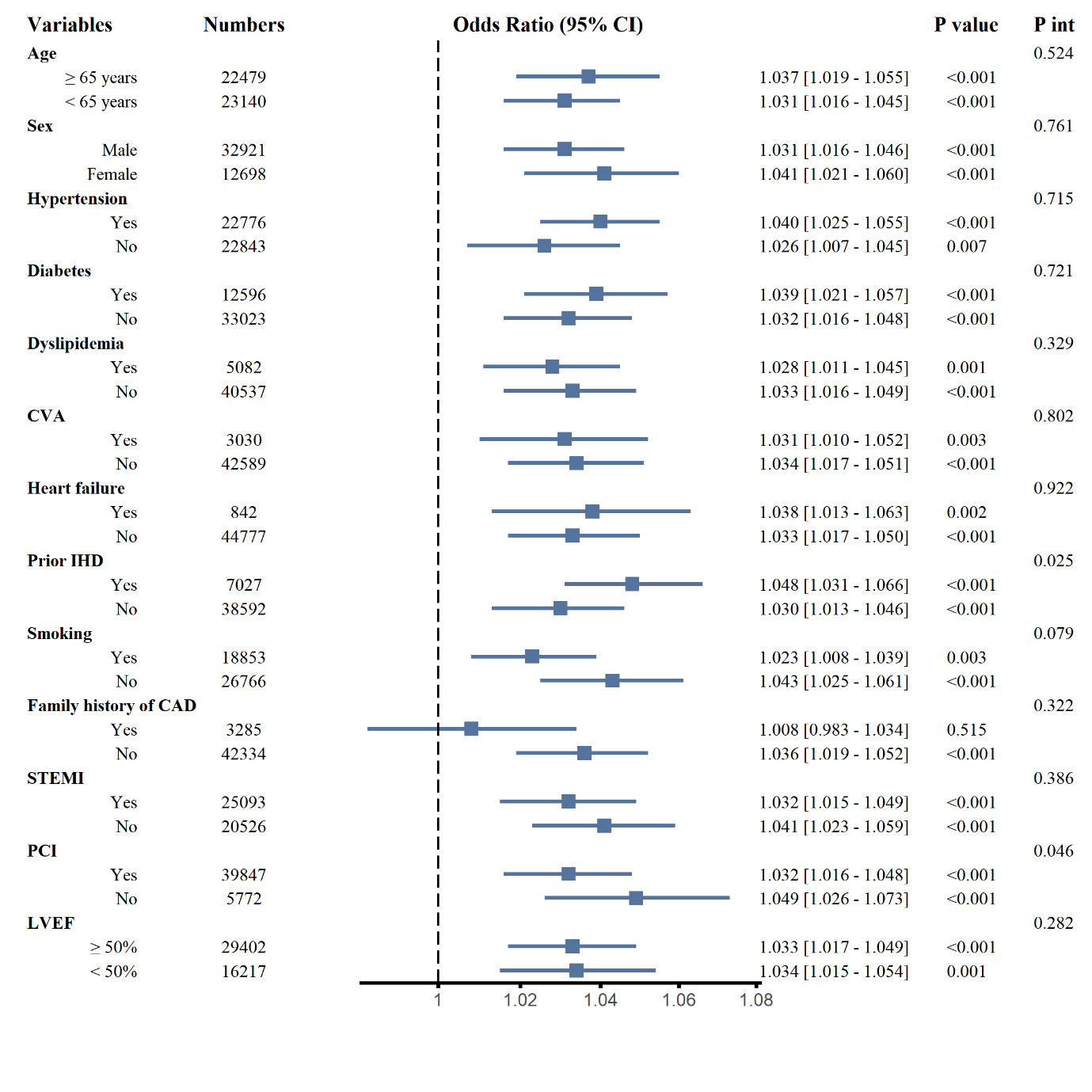
CI = confidence interval, CVA = cerebrovascular accident, IHD = ischemic heart disease, CAD = coronary artery disease, STEMI = ST-elevation myocardial infarction, PCI = percutaneous coronary intervention, LVEF = left ventricular ejection fraction.
